# Kangaroo mother care among hospitalised neonates: evaluation of the validity of duration measurement methods compared to observation linked to the OMWaNA trial in Uganda

**DOI:** 10.1186/s12887-025-05629-1

**Published:** 2025-04-09

**Authors:** Victor S. Tumukunde, Isaac Sekitoleko, Charles Opondo, Moffat Nyirenda, Cally J. Tann, Joy E. Lawn, Melissa M. Medvedev

**Affiliations:** 1 Non-Communicable Disease Programme, Medical Research Council, Virus Research Institute and LSHTM Uganda Research Unit, PO Box 49, Entebbe, Uganda; 2 Department of Infectious Disease Epidemiology and International Health, London School of Hygiene & Tropical Medicine, Keppel Street, WC1E 7HT London, UK; 3 Department of Medical Statistics, London School of Hygiene & Tropical Medicine, Keppel Street, WC1E 7HT London, UK; 4 Neonatal Medicine, University College London Hospitals NHS Trust, 235 Euston Road, NW1 2BU London, UK; 5 Department of Pediatrics, University of California San Francisco, 550 16th Street, Box 1224, 94158 San Francisco, CA USA

**Keywords:** Kangaroo mother care, Low-birth-weight, Neonate, Prematurity, Validation

## Abstract

**Background:**

Studies evaluating the impact of kangaroo mother care (KMC) on neonatal mortality and morbidity often rely on healthcare worker records or caregiver reports to measure intervention duration. However, the accuracy of these methods remains uncertain. We examined the validity of different methods of KMC duration measurement amongst neonates ≤ 2000 g in Uganda.

**Methods:**

This observational study was embedded within the OMWaNA trial, which examined the impact of KMC on neonatal mortality before clinical stability. An independent observer (considered the gold standard) monitored neonates every 2 h to confirm KMC position, using an Android tablet-based application adapted from the EN-BIRTH study. The gold standard was compared to routine healthcare workers’ charting and caregiver diary reports of KMC.

**Results:**

Among 222 caregiver-newborn pairs, 219 initiated KMC. The mean daily KMC duration recorded by the gold standard was 8·4 h (SD 3·5). Healthcare workers reported an average of 8·5 h (SD 4·0), while caregivers reported 10·4 h (SD 3·8). The mean difference was 0·2 h less for healthcare workers (95% CI -0·3 to 0·6) and 1·7 h more for caregivers (-2·1 to -1·3) compared to the gold standard. Agreement rates for individual KMC episodes were 55·2% (95% CI 54·4–55·9) for healthcare workers and 58·2% (57·2–59·0) for caregivers. Participants with a helper (substitute KMC provider) had longer daily duration compared to those without (mean difference 1·89 h [0·89 − 2·84]; *p* < 0·001).

**Conclusion:**

Healthcare worker records provide a reasonably accurate estimate of KMC duration at the population level, supporting the integration of KMC indicators into national health information systems to facilitate monitoring and evaluation. The presence of a helper increases KMC duration, underscoring the need for research to identify strategies to increase family involvement.

## Research in context

***Evidence before this study***: Kangaroo mother care (KMC) improves survival of low birthweight neonates, with longer daily KMC duration linked to lower mortality risk. However, analyses of KMC duration remain unclear, partly due to the variability and unknown accuracy of measurement methods. Studies have traditionally relied on healthcare workers’ records or caregiver reports to assess KMC duration. We searched PubMed, without language restrictions, for published studies on validation of KMC duration measurement methods from Jan 1, 1990, to 21 November 2022, using the following search terms: “kangaroo mother care” [MeSH], or “care method, kangaroo mother” [MeSH], or “skin-to-skin contact” [MeSH], or “skin-to-skin care” [MeSH], and “monitoring,” or “ measurement,” or “duration”. We found that the majority of KMC studies relied on caregivers (54%) and healthcare worker records (31%) to measure KMC duration, with no reported validation of the methods.

### The added value of this study

This study is the first published validation of the accuracy of KMC duration measurement methods, comparing healthcare worker records and caregiver reports against direct observation (considered the gold standard) using a time-stamped app. The findings demonstrate that, while healthcare worker records closely match the gold standard with a minimal mean difference of 0·2 h lower, caregiver reports tend to overestimate KMC duration by an average of 1·7 h. The study also highlights the benefit of having a helper (substitute KMC provider), with an additional 1·89 to 2·03 h of KMC daily, depending on adjustments for maternal factors.

### Implications of all the available evidence

The reasonable accuracy of healthcare worker recording of KMC duration supports wider use, potentially in national health information systems, facilitating more reliable monitoring and evaluation of the quality of KMC implementation. However, the lower agreement rates suggest the need for improved training and standardisation of reporting tools to enhance KMC duration measurements by healthcare workers. Caregiver reports are useful for tracking KMC coverage but should be interpreted cautiously when assessing KMC duration, especially in the absence of healthcare worker records. Our research also contributes more robust evidence to underscore the value of involving family members to increase the duration and effectiveness of this life-saving intervention.

## Introduction

Globally, 2·3 million neonatal deaths (first 28 days after birth) were estimated to occur in 2022 [1]. Small vulnerable newborns (SVN), including those born preterm and small-for-gestational-age, account for more than half of neonatal deaths, and have an increased risk for post-neonatal mortality and growth failure [2, 3]. Mortality risk is highest in low- and middle-income countries (LMIC) due to gaps in coverage and quality of neonatal care [4]. Improving the care of small and sick neonates in hospitals is crucial to accelerating neonatal survival and meeting Sustainable Development Goals (SDGs) by 2030 [2, 5].

Kangaroo mother care (KMC) involves early and prolonged skin-to-skin contact (SSC), promotion of exclusive breastfeeding or breastmilk feeding, and follow-up after discharge. KMC is associated with decreased mortality, sepsis, hypothermia, hypoglycemia, and length of hospital stay compared to conventional care among clinically stable neonates [6–8]. A meta-analysis of three trials of KMC initiated before stabilisation showed a 19% relative reduction in neonatal mortality at 28 days [9]. KMC is recommended by the World Health Organisation (WHO) to be initiated as soon as possible after birth in all neonates weighing < 2500 g (g) [10].

Longer durations of KMC are crucial for achieving positive health outcomes [8, 11, 12]. Research indicates that KMC only reduces mortality in stable neonates when provided for 20 h or more daily, according to a Cochrane review (2016) [6]. A more recent review reported a significant reduction in mortality at 28 days when the daily duration was at least 8 h [12]. Conversely, another systematic review highlighted that some benefits of KMC are lost when the KMC duration is 2 h or less [13]. The OMWaNA trial in Uganda showed that neonates in the intervention group who received a median of 12–24 h of KMC per day had a lower risk of mortality at 7 days and 28 days compared to those receiving < 12 h per day [9]. Although the WHO recommends 8–24 h of KMC daily, this guideline is based on a systematic review that highlighted a lack of sufficient data on the optimal duration of KMC [12]. The absence of reliable and validated methods to measure KMC duration complicates the interpretation of evidence from meta-analyses that combine studies with varying KMC measurement methods [6].

Barriers to a higher duration of KMC have been reported at both the health facility and community levels. Studies have found that lack of beds and space, privacy issues, inadequate caregiver education, insufficient staff and monitoring devices, and difficulties motivating mothers to devote time were common barriers to KMC continuity in health facilities [14–16]. Maternal factors, such as fatigue, depression, and postpartum pain, especially after a cesarean section, may reduce uptake and the time spent in the KMC position [17]. Women may find long hours of KMC challenging, impeding sleeping and eating, or after discharge, affecting time for household activities [17].

Our recent scoping review found 54 studies reporting on KMC duration. Of these, the majority of studies (29, 54%) used caregiver reports and a few (17, 31%) used healthcare worker records to measure the duration of the intervention [18]. However, evidence on the validity of these methods to accurately measure KMC duration is lacking. One previous study in Tanzania, Bangladesh, and Nepal validated the coverage indicator of KMC and measured duration using a time-stamped app but did not validate the measurement of duration [19]. Although it is plausible that longer durations of KMC improve health outcomes amongst neonates, the evidence remains incomplete without more rigorously validated methods for measuring the duration of KMC.

This study aimed to evaluate the validity of different methods for measuring KMC duration, compared to the gold standard of direct observation, among admitted newborns weighing ≤ 2000 g in Uganda.

## Methods

### Study design, settings, and population

This was an observational validation study embedded in the OMWaNA trial, a randomised, controlled trial examining the effect of KMC initiated before clinical stability on neonatal mortality, relative to standard care, in Uganda [20]. The trial recruited between October 2019 and July 2022 in five hospitals across Uganda. Neonates recruited to the trial between October 2021 and July 2022 at the largest trial site, Kawempe National Referral Hospital in Kampala, were included in this KMC duration measurement validation sub-study [9]. Participants included singleton, twin, or triplet (if triplet pregnancy resulted in the demise of ≥ 1 fetus) neonates born weighing 700–2000 g who were randomised to the intervention (KMC) arm of the trial, and their caregivers. Neonates with life-threatening instability (defined as oxygen saturation < 88% while on oxygen support, and ≥ 1 of heart rate < 100 or > 200 beats/minute, respiratory rate < 20 or > 100 breaths/minute apnoea requiring bag-mask ventilation), jaundice requiring immediate treatment, active seizures, or major congenital malformation were excluded from the study.

### Study procedures

KMC was initiated as soon as possible following recruitment into the trial. Neonates were placed onto the exposed chest of their caregiver skin-to-skin using a KMC wrap. Before placing neonates in the KMC position, a study nurse or medical officer demonstrated to caregivers how to perform KMC, breastfeed, and feed expressed breastmilk. An independent observer (considered the gold standard) monitored neonates every 2 h around the clock to record time-stamped data documenting if they were in the KMC position, as done in the EN-BIRTH KMC coverage indicator validation study [19]. They also documented the KMC provider (mother or substitute provider) and the reason for not performing KMC if it was not being practiced at the time of observation. Data were collected using a custom-built Android tablet-based software application adapted from the EN-BIRTH study [19]. During routine nursing observations (every three hours), a study nurse (healthcare worker record) documented whether neonates were in the KMC position. Caregivers were provided with a “diary” in terms of a paper chart and a pen, and a study nurse demonstrated how to record the start and end time of each episode of KMC (caregiver report). Illiterate caregivers were assisted to report by a literate caregiver participant. Results are reported following the STROBE statement checklist for cross-sectional studies (appendix 1).

### Sample size

The sample size for the sub-study was 222 caregiver-baby pairs. This sample size provided 80% power to detect a difference of at least 0·96 h in the mean daily duration of KMC, assuming an expected daily mean of 8 h and a standard deviation (SD) of the difference in any pairwise comparison of 5·1 h.

### Data analysis

Participants’ social demographic characteristics were summarised using frequencies and proportions. We calculated the healthcare worker-recorded and caregiver-reported duration of KMC and compared them with the independent observer’s documented KMC duration as means. To assess the accuracy of population-level performance, we independently calculated and compared the gold standard observation with the healthcare worker record and caregiver-reported KMC duration for all mother-baby pairs using Bland-Altman plots. At the individual level, point observations/records of KMC practice for both methods were classified as either ‘yes’ if the newborn was observed to be, or reported by the caregiver to have been in KMC position, and ‘no’ if not. These were then compared against the gold standard. Validity measures using the “diagnostic test” approach were calculated using two-way tables, excluding any missing pairwise data. Sensitivity, specificity, and positive predictive values were calculated for the two methods. A logistic regression model was applied to estimate the difference in KMC duration between the participants with a helper (substitute KMC provider) and those without. Stata version 18 (College Station, TX, USA) was used for all quantitative analyses.

## Results

A total of 222 mother-baby pairs were enrolled, among which 219 initiated KMC. Three babies never started KMC due to worsening clinical conditions. About half of the newborns were male (Table 1). The mean gestational age at screening was 32 weeks (SD 2·5; 95% [CI] 26–38) and the mean birthweight was 1·5 kg (SD 0·3; 95% [CI] 0·8 − 2·0. Most women were aged 23–34 years of age and were married or cohabiting (Table 1).

**Table 1:** Characteristics of newborns and their mothers

Variable	% (*n*/*N*)
**Mothers**	
**Age (years)**	
< 20	10% (22/219)
20–34	80.8% (177/219)
> 35	7.3% (16/219)
**Mode of delivery**	
Normal spontaneous vaginal delivery	88.7% (196/221)
Cesarean delivery	9.9% (22/221)
Forceps or vacuum-assisted vaginal delivery	1.4% (3/221)
**Employment status**,** n (%)**^^^	
Formal employment	20.4% (45/221)
Informal employment	34.4% (76/221)
Unpaid labour	45.2% (100/221)
**Neonates**	
**Male sex**	50.7% (111/219)
Gestational age^*^at screening (weeks),** mean (SD)**	32 (2.5)
**Birthweight (kg)**,** mean (SD)**	1.5 (0.3)
**Birthweight distribution (g)**,** n (%)**	
700 to < 1000	3.2% (7/220)
1000 to < 1500	35% (77/220)
1500 to 2000	61.8% (136/220)

^*^Gestational age calculated by Ballard score. ^^^Formal employment includes work for the government, the private sector, or non-governmental organizations. Informal employment includes work for private households, self-employment, and work on a farm or with livestock. Unpaid labour includes unemployment, student, homemaker, and retirement

Caregivers reported 14,031 (77·5%) point observation episodes in which the newborn was in the KMC position while the other time observations the newborn was not. Healthcare workers recorded 9,574 (53·1%) point observation episodes in which the newborn was in the KMC position. The independent observer reported 9,321 (51·1%) point observation episodes in which the newborn was in the KMC position. The KMC provider was a substitute caregiver (not the mother) in 2,455 (26·3%) observations by the independent observer in which the newborn was in the KMC position. The majority of substitute KMC providers were female relatives of the newborn, including auntie (51·6%) followed by grandmother (22·5%). Others included siblings (11·2%), fathers (11·1%), uncles (6·5%), and friends of the mother (0·7%).

For 8,905 observations by the independent observer, newborns were not in the KMC position. The reasons for not doing KMC were recorded for 8,250 (92·6%) observations. Caring for the newborn (including feeding, cleaning, and medical care) was the main reason at 59·6% followed by caregiver self-care (bathing and having meals). Others included caregiver fatigue (8·1%), caregiver doing other cores (5·1%), newborn ill-health (4·0%), and caregiver ill-health (1·5%).

**Fig. 1:**
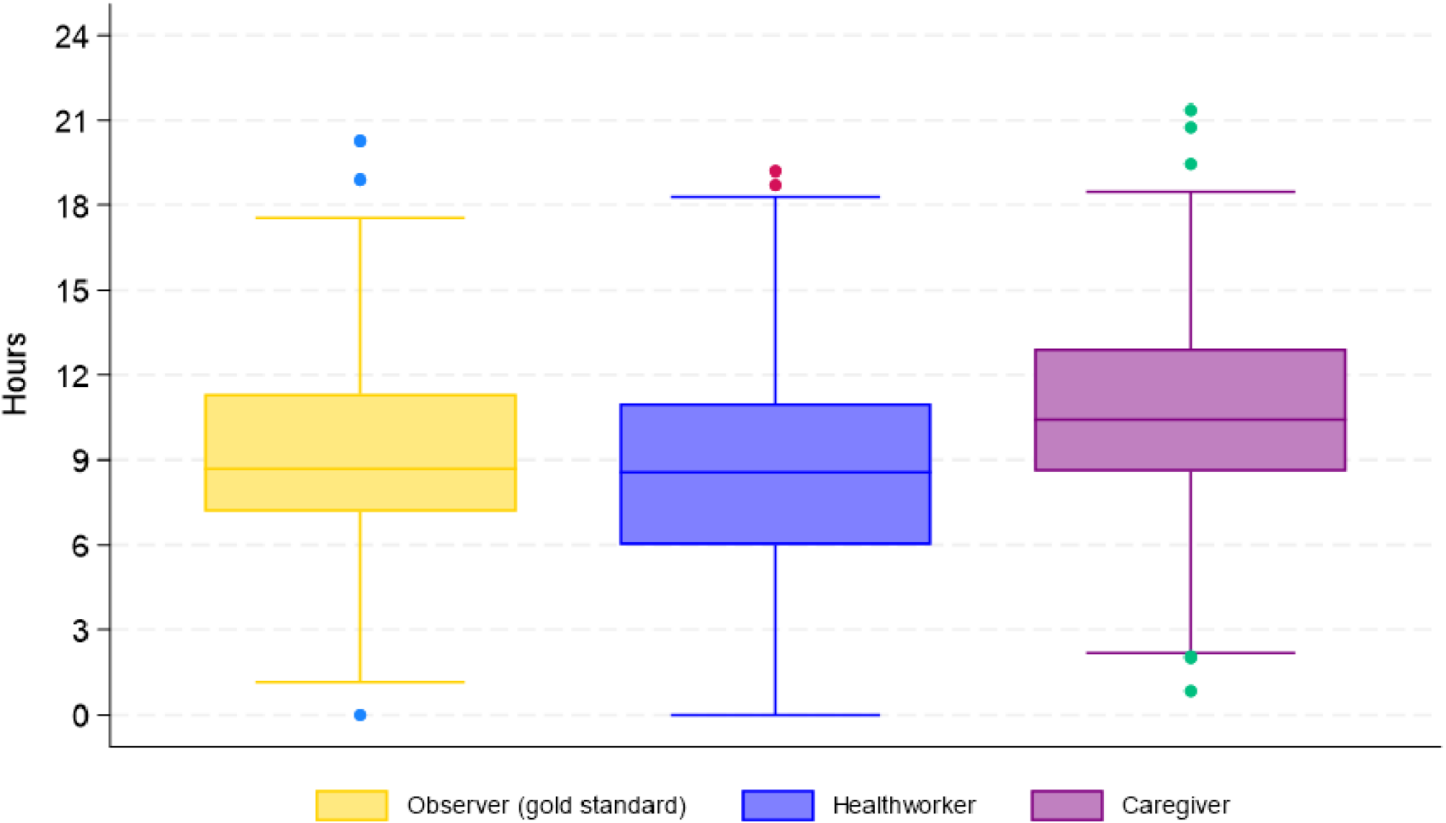
Mean daily KMC duration comparing observation with healthcare worker record and caregiver report

The mean cumulative KMC duration reported by independent observers was 67·8 h (SD 64·6), with a mean daily duration of 8·4 h (SD 3·5). Caregivers reported a mean cumulative KMC duration of 82·6 h (SD 68·6) and a mean daily duration of 10·4 h (SD 3·8), while healthcare workers recorded a mean cumulative KMC duration of 60·5 h (SD 34·4) and a mean daily duration of 8·5 h (SD 4·0) (Table 2; Fig. 1).

**Table 2:** Cumulative and daily mean duration of KMC and validation test outcomes

	Cumulative duration of KMC (hours), mean (SD)	Cumulative difference between methods and observer (hours), mean (95% CI)	Daily KMC duration (hours), mean (SD)	Daily difference between methods and observer (hours), mean (95% CI)	Sensitivity (95% CI)	Specificity (95% CI)	Positive predictive value (PPV)	Percent agreement^*^
**Observer (gold standard)**	67·8 (64.4)	-	8·4 (3·5)	-	-	-	-	-
**Healthcare worker record** ^**†**^	60·5 (34.4)	9·5 (3.5– 15.5)	8·5 (4·0)	0·2 (-0.3 - 0.6)	52·6 (51·9-53·3)	59·1 (58·3-59·8)	57·6	55·7%
**Caregiver report** ^**^**^	82·6 (68.6)	-14·1 (-17.6 - -10.6)	10·4 (3·8)	-1·7 (-2.1 - -1.3)	85·1 (84·5-85·6)	30·5 (29·9-31·2)	56·4	58·6%

CI=confidence interval. SD=standard deviation. ^*^Percent agreement= (true positives + true negatives)/n. ^†^Healthcare worker record data were missing for 185 (1.0%) observations. ^^^Caregiver report data were missing for 120 (0.7%) observations

Using the mean cumulative KMC duration to assess the level of agreement between the methods, the mean difference between independent observer and healthcare worker record was 9·5 (95% CI 3·5 to 15·5), implying that healthcare workers recorded KMC duration 9·5 h shorter throughout observation. The mean difference between independent observer and caregiver reports was − 14·1 (95% CI -17·6 to -10·6), meaning that caregivers reported KMC duration 14 h longer throughout observation.

Using the mean daily KMC duration to assess the level of agreement between the methods, the mean difference between independent observer and healthcare worker records was 0·2 (95% CI -0·3 to 0·6; Fig. 2A). The mean difference between the independent observer and caregiver report was − 1·7 (95% CI -2·1 to -1·3; Fig. 2B).

The percentage agreement between independent observers and the two measurement methods was 55·7% for healthcare workers and 58·6% for caregivers (Table 2). Healthcare worker records had a sensitivity of 52·6% and a specificity of 59·1%, while caregiver reports had a sensitivity of 85·1% and a specificity of 30·5%. The two methods had low positive predictive values of 57·6% and 56·4% for healthcare worker records and caregiver reports, respectively.

About two-thirds (*n* = 149, 67·1%) of participants had a helper during the hospital stay who acted as a substitute KMC provider in the place of the mother, accounting for 2,455 (26·3%) observations. Participants with a substitute KMC provider had a mean daily KMC duration of 9·8 h (SE 0·3) compared to 7·9 h (SE 0·4) for those who did not have a substitute KMC provider (mean difference 1·9 h; 95% CI 0·9 − 2·8; *p* < 0·001). After controlling for maternal age, parity, marital status, and employment category, the adjusted mean difference in daily KMC duration increased to 2·0 h (95% CI 1·0–3·0; *p* < 0·001).

**Fig. 2A:**
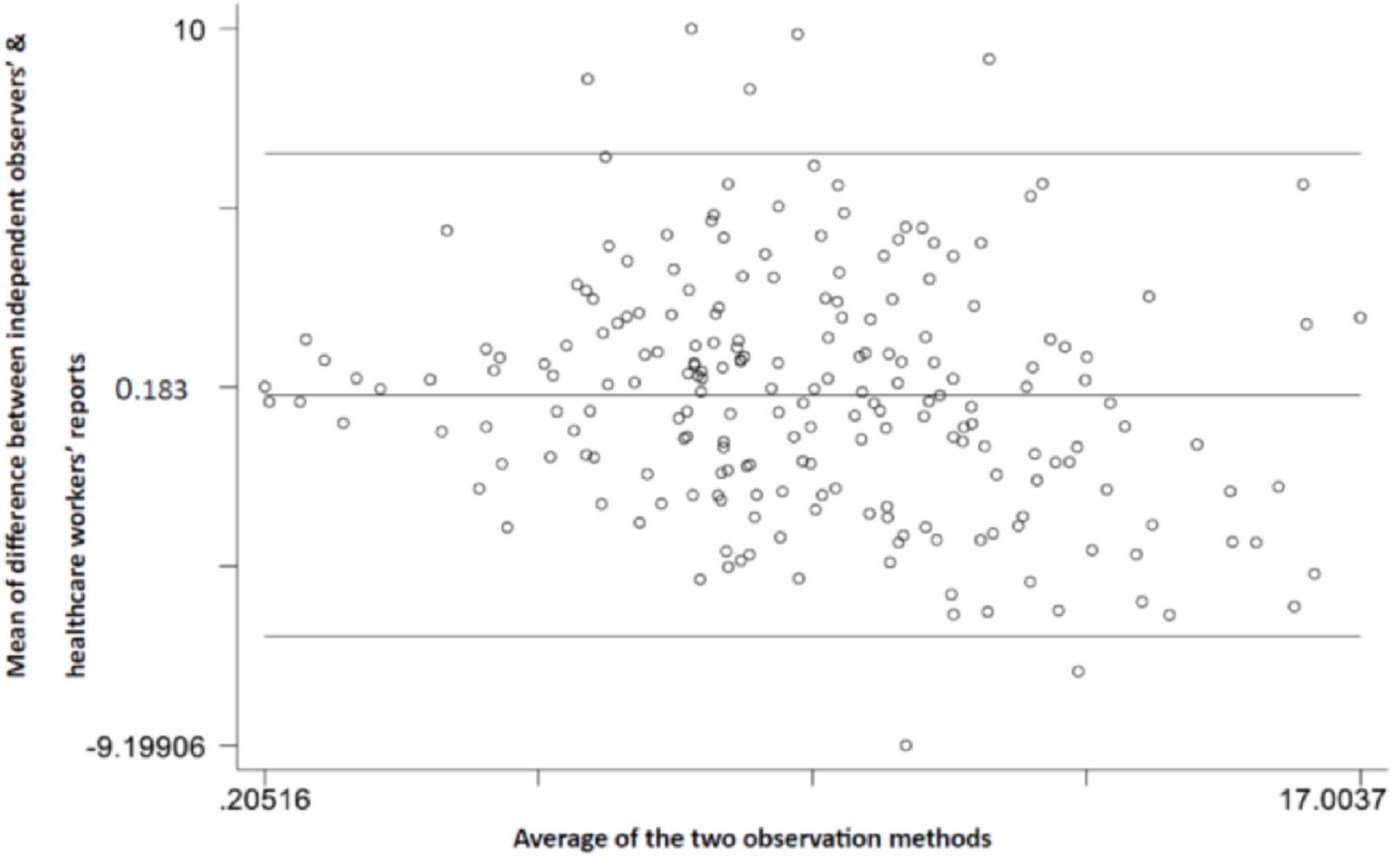
Mean difference in daily KMC duration between independent observers’ and healthcare workers’ records

**Fig. 2B:**
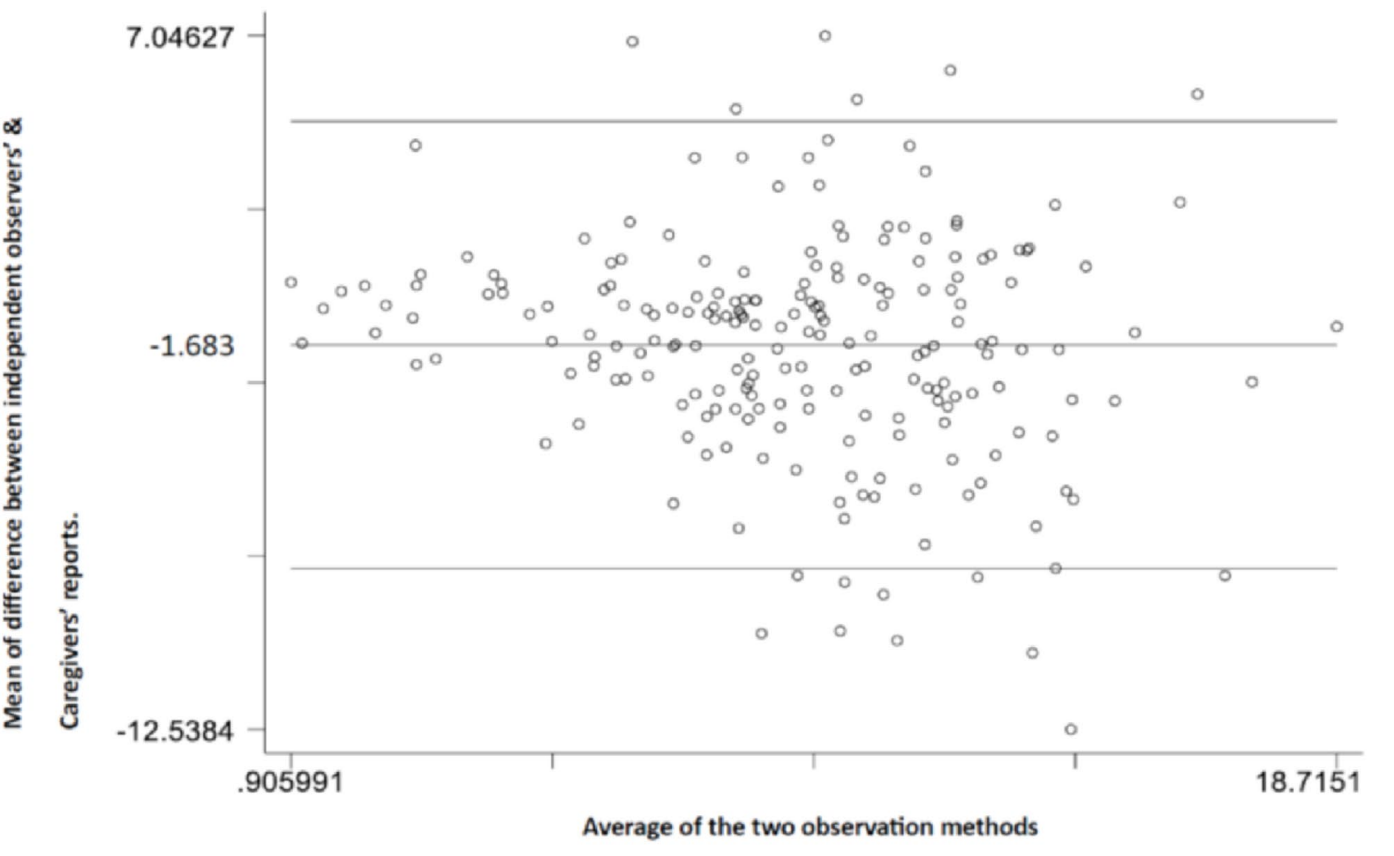
Mean difference in daily KMC duration between independent observers’ and caregivers’ reports

## Discussion

This is the first study to test the validity of healthcare worker records and caregiver reports for KMC duration measurement, compared against independent observers as a “gold” standard using a time-stamped app. Our findings demonstrate that while healthcare worker records closely match the gold standard with a minimal mean difference of 0·2 h lower, caregiver reports tend to overestimate KMC duration by an average of 1·7 h. The study also highlights the significant impact of having a substitute KMC provider, with participants benefiting from an additional 1·9 to 2·0 h of KMC daily.

Our results suggest that healthcare worker records can provide a relatively accurate estimate of KMC duration at the population level. These records could be integrated into national health information systems to support the monitoring and evaluation of KMC implementation. Findings from the EN-BIRTH study also supported this, showing that routine hospital KMC registers have the potential to track intervention coverage in KMC wards [19].

Healthcare worker record was only slightly lower than the gold standard at the population level but did have low sensitivity (53%) and specificity (59%) at the individual level. Despite this, healthcare worker records showed reasonable point discrepancies with the gold standard at the individual level. To improve practice, incorporating more focused KMC-specific training or dedicated recording time may help enhance the accuracy of individual-level data. A study in Malawi found that healthcare workers tend to underestimate outcome measurements, and this practice increases over time [21]. Another study reported evidence of over-reporting of newborn quality of care indicators compared to the gold standard [22]. Other studies have also reported low accuracy in healthcare workers’ documentation of medical records, especially for interventions rather than clinical outcomes [23]. However, training healthcare workers in data management has been shown to improve both the completeness and accuracy of data [24]. This highlights the importance of training healthcare workers in KMC data monitoring as part of routine data collection to support the scaling up of the intervention.

The high sensitivity (85%) of caregiver reports in this study may be due to social desirability bias, where caregivers tend to present a more favourable image [25]. Similar findings were observed in a study validating KMC coverage through direct observation and exit interviews, which also reported high sensitivity but did not assess KMC duration [26]. Generally, maternal self-reports have been noted to show higher sensitivity for events occurring immediately after childbirth, which aligns with our findings [27]. However, caregiver reports in this study tended to overestimate the average daily KMC duration by about 1·7 h. This implies that caregiver reports can be useful for tracking KMC coverage but should be interpreted with caution, particularly when assessing the duration of KMC. Healthcare systems should consider incorporating cross-verification mechanisms, such as routine checks or combining caregiver reports with healthcare worker records, to ensure more accurate reporting.

About 67% of mother-baby pairs had a helper in the hospital who acted as a substitute KMC provider. These participants received an additional 1·9 to 2·0 h of KMC daily, with an adult female relative being the main substitute KMC provider. Similarly, previous studies have identified grandmothers as key family support in hospitals [19, 28]. Research has shown that having a family member present helps maintain the continuity of KMC and provides psychological support to mothers [29, 30]. The involvement of family members, particularly female relatives, is likely associated with longer KMC durations. However, there is no existing research that quantifies the additional KMC hours provided by family members apart from the findings of this study. Since longer KMC duration is linked to reduced neonatal mortality risk, further research is needed to identify strategies to increase KMC duration through family involvement.

This study has strengths, including the provision of novel data on the validity of healthcare worker records and caregiver reports for KMC duration measurement and the use of a time-stamped software application to improve data capture. However, our study also has some limitations. The frequency of independent observer measurements, at 2-hour intervals, may have introduced a bias into the calculated agreement rates, as KMC duration may have varied between these observations. Continuous observation has not been considered feasible in other studies. Continuous video recording has been used previously in observational skin-to-skin studies; however, this has largely been for short periods of observation, for example, to examine the neonatal response to noxious stimuli like heel prick procedures [31]. Continuous video recording could be considered as an alternative reference standard for this study against which the commonly used methods in KMC studies could be validated. However, continuous video recording of KMC in an open ward care environment presents challenges around informed consent including limitations of anonymity, and the recording of non-research related activities of participants and non-participants receiving care in the mother-NICU [32]. Another option could be an electronic device allowing continuous contact assessment. Future studies could investigate ways to implement healthcare worker records and improve accuracy, such as training and standardisation of tools. These could be tested against alternative objective measures of KMC duration, such as innovative electronic position monitoring.

Integration of KMC indicators into national health information systems is a feasible and essential step for improving the monitoring and evaluation of KMC programs. Given that healthcare worker records provide reasonably accurate estimates of KMC duration at the population level, policies should prioritize the routine collection and integration of this data. Additionally, encouraging active family involvement, particularly from female relatives, can significantly extend KMC duration, thereby reducing neonatal mortality. Healthcare facilities should adopt policies that facilitate family support in KMC, creating a comprehensive approach to improving infant health outcomes. Further research is needed to develop strategies that enhance KMC duration, particularly through the involvement of family members. Investigating ways to improve the accuracy of healthcare worker records, such as through training and standardization of data collection tools will be crucial. These methods could be tested against objective measures of KMC duration, including innovative technologies like electronic position monitoring, to validate and improve program monitoring and effectiveness.

The healthcare worker record on average provides a reasonably accurate estimate of KMC duration at the population level, highlighting the feasibility of integrating KMC indicators into the national health information systems and could facilitate the monitoring and evaluation of KMC implementation with quality and lead to higher impact.

## Data Availability

The data supporting the results reported in this manuscript is stored on the Medical Research Council (MRC) and London School of Hygiene and Tropical Medicine (LSHTM) Uganda Research Unit secure servers. This data is available on request to the data manager through the corresponding author.

## Appendix 1

**Table:** STROBE statement: checklist of items to be included in reports of observational studies

		Item No	Recommendation
**Title and abstract**		1	(*a*) Indicate the study’s design with a commonly used term in the title or the abstract
			(*b*) Provide in the abstract an informative and balanced summary of what was done and what was found
**Introduction**			
Background/rationale		2	Explain the scientific background and rationale for the investigation being reported
Objectives		3	State specific objectives, including any prespecified hypotheses
**Methods**			
Study design		4	Present key elements of study design early in the paper
Setting		5	Describe the setting, locations, and relevant dates, including periods of recruitment, exposure, follow-up, and data collection
Participants		6	(*a*) *Cohort study*—Give the eligibility criteria, and the sources and methods of selection of participants. Describe methods of follow-up *Case-control study*—Give the eligibility criteria, and the sources and methods of case ascertainment and control selection. Give the rationale for the choice of cases and controls *Cross-sectional study*—Give the eligibility criteria, and the sources and methods of selection of participants
			(*b*) *Cohort study*—For matched studies, give matching criteria and number of exposed and unexposed *Case-control study*—For matched studies, give matching criteria and the number of controls per case
Variables		7	Clearly define all outcomes, exposures, predictors, potential confounders, and effect modifiers. Give diagnostic criteria, if applicable
Data sources/ measurement		8*	For each variable of interest, give sources of data and details of methods of assessment (measurement). Describe comparability of assessment methods if there is more than one group
Bias		9	Describe any efforts to address potential sources of bias
Study size		10	Explain how the study size was arrived at
Quantitative variables		11	Explain how quantitative variables were handled in the analyses. If applicable, describe which groupings were chosen and why
Statistical methods		12	(*a*) Describe all statistical methods, including those used to control for confounding
			(*b*) Describe any methods used to examine subgroups and interactions
			(*c*) Explain how missing data were addressed
			(*d*) *Cohort study*—If applicable, explain how loss to follow-up was addressed *Case-control study*—If applicable, explain how matching of cases and controls was addressed *Cross-sectional study*—If applicable, describe analytical methods taking account of sampling strategy
			(*e*) Describe any sensitivity analyses
**Results**			
Participants	13*	(a) Report numbers of individuals at each stage of study—eg numbers potentially eligible, examined for eligibility, confirmed eligible, included in the study, completing follow-up, and analysed	
		(b) Give reasons for non-participation at each stage	
		(c) Consider use of a flow diagram	
Descriptive data	14*	(a) Give characteristics of study participants (e.g. demographic, clinical, social) and information on exposures and potential confounders	
		(b) Indicate number of participants with missing data for each variable of interest	
		(c) *Cohort study*—Summarise follow-up time (e.g., average and total amount)	
Outcome data	15*	*Cohort study*—Report numbers of outcome events or summary measures over time	
		*Case-control study—*Report numbers in each exposure category, or summary measures of exposure	
		*Cross-sectional study—*Report numbers of outcome events or summary measures	
Main results	16	(*a*) Give unadjusted estimates and, if applicable, confounder-adjusted estimates and their precision (e.g., 95% confidence interval). Make clear which confounders were adjusted for and why they were included	
		(*b*) Report category boundaries when continuous variables were categorized	
		(*c*) If relevant, consider translating estimates of relative risk into absolute risk for a meaningful time period	
Other analyses	17	Report other analyses done—eg analyses of subgroups and interactions, and sensitivity analyses	
**Discussion**			
Key results	18	Summarise key results with reference to study objectives	
Limitations	19	Discuss limitations of the study, taking into account sources of potential bias or imprecision. Discuss both direction and magnitude of any potential bias	
Interpretation	20	Give a cautious overall interpretation of results considering objectives, limitations, multiplicity of analyses, results from similar studies, and other relevant evidence	
Generalisability	21	Discuss the generalisability (external validity) of the study results	
**Other information**			
	22	Give the source of funding and the role of the funders for the present study and, if applicable, for the original study on which the present article is based	

*Give information separately for cases and controls in case-control studies and, if applicable, for exposed and unexposed groups in cohort and cross-sectional studies

## List of Abbreviations

CPAP: Continuous positive airway pressure
eCRF: Electronic case report form
eKMC: Early kangaroo mother care (clinical trial)
iKMC: Immediate Kangaroo mother care (clinical trial)
G kg: Grams Kilograms
GCP: Good Clinical Practice
HR: Heart rate
ICH: International Council for Harmonization
IRB: Institutional Review Board
IQR: Interquartile range
IV: Intravenous
KMC: Kangaroo mother care
LBW: Low birth weight
LMIC: Low- and middle-income countries
LSHTM: London School of Hygiene & Tropical Medicine
MRC: Medical Research Council
OMWaNA: Operationalizing kangaroo Mother care among low birth-weight Neonates in Africa (clinical trial)
RCT: Randomized controlled trial
REC: Research Ethics Committee
SD: Standard deviation
SDG: Sustainable Development Goal
SSC: Skin-to-skin care
UCSF: University of California San Francisco
UVRI: Uganda Virus Research Institute
UPA: Uganda Paediatric Association
UNICEF: United Nations Children’s Fund
NEST 360^o^: Newborn Essential Solutions and Technologies (NEST 360°)
WHO: World Health Organization
EN-BIRTH: Every Newborn-Birth Indicators Tracking in Hospitals
NSCU: Neonatal Special Care Unit
NMR: Neonatal Mortality Rate
U5MR: Under-five Mortality Rate
